# Treatment Engagement of Psychotic Patients with a Mobile Mental Health Unit in Rural Areas in Greece: A Five-Year Study

**DOI:** 10.1155/2013/613956

**Published:** 2013-10-03

**Authors:** Vaios Peritogiannis, Athina Tatsioni, Nefeli Menti, Aikaterini Grammeniati, Vassiliki Fotopoulou, Venetsanos Mavreas

**Affiliations:** ^1^Mobile Mental Health Unit of the Prefectures of Ioannina and Thesprotia, Society for the Promotion of Mental Health in Epirus, 45445 Ioannina, Greece; ^2^Department of Internal Medicine, University of Ioannina School of Medicine, 45500 Ioannina, Greece; ^3^Department of Psychiatry, University of Ioannina School of Medicine, 45500 Ioannina, Greece

## Abstract

*Objectives*. Treatment of psychotic disorders is impended by high rates of disengagement from mental health services and poor adherence to antipsychotic medication. This study examined the engagement rates of psychotic patients with a community mental health service during a 5-year period. *Methods*. The Mobile Mental Health Unit of Ioannina and Thesprotia (MMHU I-T) delivers services in remote, rural, mountainous areas using the resources of the primary care system. Clinical and demographic information for patients with a diagnosis of schizophrenia and related psychoses was obtained from the medical records of our unit. *Results*. A total of 74 psychotic patients initially engaged in treatment with our unit. In half of cases treatment was home-based. With the exclusion of patients who died or discharged, engagement rates were 67.2%. Statistical analysis was performed for 64 patients, and no differences were found between engaged and disengaged patients regarding clinical and demographic parameters. All engaged patients regularly refilled their antipsychotic prescriptions. *Conclusion*. Engagement rates in our study were comparable to previous research, involving urban settings and shorter follow-up duration. Community mental health teams may ensure treatment continuation for psychotic patients in deprived, remote areas. This is important for low-income countries, affected by economic crisis, such as Greece.

## 1. Introduction

Schizophrenia and related disorders are chronic and disabling and have a major impact on the person, the family, and the society in general. Continuity of care is considered essential in the effective management of such long-term disorders by service users, clinicians, and health care policy makers [1]. Previous studies have suggested that continuity of care is positively associated with health outcomes among persons with severe mental illness [2]. More recently, continuity of care was linked to better social functioning of people with chronic psychotic disorders [3]. However, despite the availability of effective pharmacological and psychosocial treatments for the management of psychotic disorders, treatment is impended by high rates of disengagement from mental health services and poor adherence to antipsychotic medication. Treatment discontinuation has severe consequences for the patients, such as relapse, hospitalization, homelessness, suicide, and violence [4, 5]. This may be particularly the case of psychotic patients living in poor and deprived remote rural areas who may not receive appropriate mental health care.

Evidence from Eastern European countries suggests that access to mental health services is limited for several reasons including location, age, employment status, and socioeconomic status. In the above countries more services are available in urban areas compared to rural settings [6]. Similarly, in Greece psychiatric patients living in rural, remote, and deprived areas do not receive adequate mental health care [7]. In Epirus, Northwestern Greece, the Mobile Mental Health Unit of the prefectures of Ioannina and Thesprotia (MMHU I-T) was established in 2007 with the aim of delivering services to such areas. The MMHU I-T is now at the sixth year of operation. The basic principles of its operation as well as its impact on psychotic relapses and hospitalization have been reported elsewhere [8]. The purpose of the present study was to evaluate the engagement rates of chronic psychotic patients who were treated in our service during a five-year period. Treatment engagement and medication adherence are essential components of community-based mental health care of people suffering from psychotic disorders [9].

## 2. Materials and Methods

### 2.1. Service Description

The MMHU I-T delivers generic mental health care for a population of approximately 100.000 inhabitants living in rural, remote areas. It should be noted that due to geographical conditions (the whole area is mountainous) access to mental health services is limited, particularly in winter. Our unit uses the resources of the primary health care system and visits patients at home when necessary. In rural areas of Greece there is a well-established primary health care system organized in local units, such as health centers and regional medical offices [10]. The multidisciplinary team provides evidence-based care for patients with a mental disorder, including pharmacotherapy and psychotherapeutic interventions. Enhancement of patients' social skills is a major priority as well as education and support for their families. The assumption of activities that promote mental health and the implementation of educational programs in the community are also basic pursuits [8].

### 2.2. Study Design and Participants

**Figure 1:**
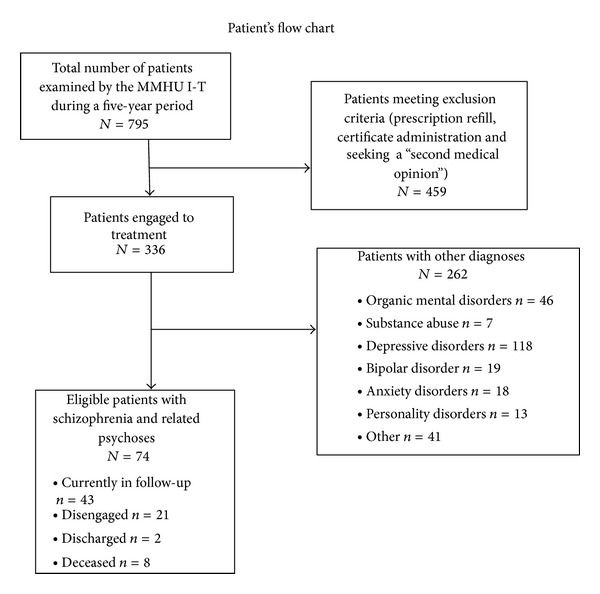


Medical records of all the patients who have been examined by our unit during a 5-year period (from March 2007 to March 2012) were retrospectively reviewed. We included all patients who had a diagnosis of schizophrenia or related psychoses and required engagement with a mental health service. We excluded patients who were referred to our unit for prescription refill and certificate administration. We also excluded patients who visited our unit because they or their families were seeking a “second medical opinion” (Figure 1).

Information regarding eligible patients with a diagnosis of schizophrenia and related psychoses was obtained, including demographic (age, gender, and living with a caregiver), and clinical information (baseline illness severity, substance abuse, and illness duration). Baseline psychopathology severity was measured with the clinical global impressions (CGI) scale. Information on patients' substance and alcohol use patterns was collected from patients' charts at the time of presentation to our service. Current alcohol and/or substance abuse was assessed according to clinician judgment, based on patients' self-reports, family accounts, and primary care medical notes. We included in the analysis only variables that have been reported in the literature as potential factors related to engagement with mental health services [11]. Insight was not measured in this study. Duration of follow-up and type of care (home-based or office-based) were also recorded. Cases of patients who died during follow-up and cases of patients who were discharged from our service were recorded as well.

### 2.3. Service Engagement Definition

Psychotic patients' service engagement was defined as regular attendance (at least 80% of the scheduled sessions) of follow-up appointments according to individual plan of care, which in most cases involved fortnightly or monthly reexamination and in some instances weekly appointments. Disengagement from our team was defined as missing the follow-up appointments for six months or longer, so as to include a few stabilized patients who wished longer follow-up intervals, such as every two or three months. Patients who attended only irregularly (i.e., less than 80% of the scheduled sessions) were rated as “disengaged,” even if intervals between their appointments were shorter than six months. By this way, none of the patients could be rated as “partially engaged” but rather as “disengaged.” Partial treatment adherence is common in psychotic disorders and has negative consequences for patients such as loss of functioning, symptoms exacerbation, and relapse [12].

All the procedures were approved by the ethical committee of the Mental Health Sector of Ioannina and Thesprotia.

### 2.4. Statistical Analysis

Data were presented as absolute numbers and percentages for binary variables and as mean with standard deviation (SD) for continuous variables. We reported our data separately for patients who were currently in follow-up and for disengaged patients. Comparisons between the two patient groups were performed using Chi-square or Fisher's exact test for binary variables and Student's *t*-test for continuous variables. All *P* values < 0.05 were considered statistically significant. The Statistical Package of the Social Sciences (SPSS 19.0) was used to perform all analyses.

## 3. Results

A total of 336 patients had been engaged in treatment with our unit during a 5-year period (from March 2007 to March 2012). Patients' diagnoses are presented in patients' flow chart (Figure 1). Regarding patients with a diagnosis of schizophrenia and related psychoses, a total of 116 individuals had been referred to our service during the 5-year period. Seventy-four psychotic patients were considered as “cases,” requiring engagement with a mental health service and meeting the study's inclusion criteria. For half of those patients (*n* = 37) care was home-based, with regular domiciliary visits. The vast majority of patients were poor, unemployed, and single (data are not presented in table). A large proportion of psychotic patients (22 patients or 29.7%) had a current or past history of alcohol or/and substance abuse. This finding is relevant because it suggests that our patient population consists of “real world” patients. A significant proportion of patients (*n* = 18 or 24.3%) were receiving a long acting oral (namely, penfluridol, a first-generation antipsychotic administrated once a week) or injectable antipsychotic formulation. Injections were given by the nursing staff of our unit. Also, injections could be given by the nursing staff of the local health centers, which also participated in patients' treatment.

Individuals who died during this period (*n* = 8) and those who were discharged from our team (*n* = 2) were excluded from the service engagement calculation. Patients who died during the 5-year period had a mean age of 63 years, which is well below (19.2%) the life expectancy of 78 years in Greece, in line with international reports [13]. The two discharged patients were placed to intermediate community facilities due to severe deterioration of their physical health. These ten patients had been engaged in our unit for a mean of 16.2 ± 13.2 months.

Forty-three out of 64 patients (67.2%) continued to regularly attend follow-up appointments, whereas 21 patients disengaged from follow-up. Patients' characteristics are presented in Table 1.

All patients, rated as service engaged, had regular refills of their antipsychotic prescriptions. Although this is only an indirect indicator of treatment adherence, this is an important finding, as antipsychotic administration is the cornerstone in the treatment of schizophrenia and other psychoses.

Treatment engagement was not found to be related to gender, age, or living with a caregiver. Several clinical characteristics of patients were examined, and commitment to follow-up was not associated with illness duration, type of care (home- or office-based), or illness severity at baseline. Notably, history of alcohol or substance abuse was not associated with service engagement in our patients. Analysis was not performed for the variables of economic status, employment, and being married, because, as mentioned above, there were very few patients who were not poor, and most were unemployed and single.

We were able to collect information for some of the disengaged patients. Four of the patients (19%) were hospitalized due to psychotic relapse, 5 patients (23.8%) continued to regularly refill their antipsychotic prescriptions from primary care physicians, and one patient moved out of the area. The majority of disengaged patients (11 or 52.4%) were not in contact with a primary care or a mental health service.

## 4. Discussion

During the five-year study period a chronic psychotic patients' engagement rate with MMHU I-T of 67.2% was found. This is comparable to previously reported rates involving mental health care delivered in the community [14]. More recently, a review of the literature involving disengagement from different mental health settings yielded similar rates, approximately 30% from the majority of services [11]. It should be noted however that studies reviewed by Simmonds et al. [14] involved an urban population where access was easier and distances shorter than in our catchment area. Furthermore, the duration of those studies ranged from 3 to 24 months, compared to our 5-year study. Moreover, they included a mixed patient population and not exclusively psychotic patients as in our study. From this aspect it could be considered that the results reported here are particularly relevant. A previous study involving an urban and rural schizophrenic patient population reported a 63% rate of contact with a community mental health service (psychiatric nurse) at 1-year follow-up in the rural setting [15].

It is interesting to note that none of the examined patient-related factors were associated with service engagement in our study. We assume that engagement with our unit may be better understood as service-related. Community-based treatment is acceptable to patients, nonrestrictive, and may promote continuity of care. MMHU I-T provides a flexible way of patient care. For example, half of the patient population in our study was receiving home-based care. There was no statistical difference for this variable between treatment engaged and treatment disengaged patients. However, due to access difficulties and economic adversities, many psychotic patients would have never been examined by our unit or would have discontinued treatment if care had been only office-based. Importantly, access in these areas and the performance of home visits are often difficult due to geographical reasons, as most of the catchment area is mountainous. 

In our study as many as 29.7% of the psychotic patients had a current or past history of alcohol or/and substance abuse. Substance abuse rates are reported to be high in psychotic patients and are associated with adverse outcome of psychotic disorders [16]. However, substance use, which is a major risk factor for treatment discontinuation, was not associated with service disengagement in this study. It might have been that such patients, who require more attention from services, were managed carefully and received comprehensive care, taking into account their special needs. It has been suggested that service characteristics impact heavily engagement, referring to practical details of everyday practice, such as organizing follow-up appointments, or to the level of the whole service organization, such as assertive community treatment [11].

 The mean duration of engaged patients' follow-up was over 30 months and may be considered satisfactory. Even patients who were disengaged had a mean follow-up period of almost 16 months. These results may suggest that treatment delivered by multidisciplinary mental health teams in rural remote areas is acceptable by chronic psychotic patients, and this may have a positive impact on the management of severe mental illness.

It should be noted that a significant proportion of patients, 24.3%, were receiving a long acting oral or injectable antipsychotic formulation, well above the rates of 10.7% previously reported for Greece and similar to those of other European countries [17]. Apart from the patients receiving a depot antipsychotic formulation, all patients had regular refills of oral antipsychotic prescriptions. Although not always reliable, this is an indirect indicator of antipsychotic adherence. Considering the high rates of psychotic patients' nonadherence to treatment [18, 19], this is an important finding suggesting that Mobile Mental Health Units in rural areas may ensure regular antipsychotic drug treatment, which is the cornerstone of the management of psychotic disorders.

Although we collected information about some of the disengaged patients, the majority (52.4%) were untraceable. We assume that these patients were unlikely to have been hospitalized in the inpatient unit of the general hospital of the area, as in such case they would have been referred back to our unit afterwards. The MMHU I-T has been successfully established within the local mental health network and is collaborated closely with the area's inpatient facility. However, some patients may have been hospitalized in private psychiatric facilities in other prefectures, but we believe that this involves only a small number of cases.

In a previous study with a 2-year follow-up period we have reported a significant reduction (30.4%) of the number of hospitalization in a small sample of 34 psychotic patients [8]. In the present study the patient sample is larger (*n* = 64) and the primary objective is the estimation of patients' service engagement and adherence to antipsychotic medication. Both of these concepts are prerequisitions for effective community-based treatment and subsequent reduction of admission rates. There are a number of issues that render a potential comparison of commitment proportion between the 2-year and 5-year cohort inapplicable. First, the study published in 2011 did not include treatment engagement as an outcome; this is the first paper, in which we address this research question. In addition, the two cohorts cannot be considered independent for all participants. There are patients who were included in the 2-year cohort but have been lost afterwards and have not been included in the 5-year cohort while other patients have remained in the 5-year cohort since the inception of the 2-year study. Therefore, a potential correlation between 2-year and 5-year results, which are referred to the same patient, cannot be excluded for certain participants. Finally, we believe that the 5-year follow-up design would be more precise than the 2-year study to address treatment engagement because of the larger sample size.

Our study had several limitations. It was designed as a retrospective noncontrolled study; and therefore potential confounders may not have been adequately assessed. Insight was not measured in this study. However, our clinical impression was that most patients lacked adequate insight throughout the follow-up period. Poor insight is common in psychotic disorders and has been found to predict treatment nonadherence [11, 20]. For the estimation of baseline illness severity we used CGI and not the more informative and detailed positive and negative syndrome scale (PANSS). Alcohol and substance use patterns were not described in detail and abuse was rated according to clinician judgment, without the use of a diagnostic interview or assessment schedule. Patients' outcome measurements were not reported here, but those who were engaged in our unit were not hospitalized during the follow-up period. Finally, our patient sample was relatively small. On the other hand, these results involve a representative population of community dwelling, moderately ill chronic psychotic patients, cared by a mobile community mental health team in rural, remote areas.

The results of this study suggest that in remote and deprived rural areas, which receive inadequate mental health care, Mobile Mental Health Units may achieve continuity of care for patients with psychotic disorders using limited resources. In the light of recent expert suggestions that generic mental health community teams may be equally effective to assertive community treatment at much lower cost [21], our results may be even more relevant, given the health effects of economic crisis in our country [22]. The generalizability of our findings may be limited by differences in the structure of the mental health system in other countries, such as insufficient number of full-time psychiatrists working in community mental health teams, organization of primary care system, and so forth. On the other hand, our study suggests that treatment delivered for psychotic patients in rural areas, even in countries largely affected by financial shortages like Greece, may be acceptable and achieve high engagement rates for psychotic patients. Conceivably, this form of treatment may be easily applicable and even more effective in Western, high-income countries.

## 5. Conclusions

In our study, rates of psychotic patients' treatment engagement were high and seem to be determined by service-related rather than patient-related factors. This is a clinically and operationally relevant finding, because it suggests that low-cost, multidisciplinary mental health teams may ensure treatment continuation for psychotic disorders, even in remote and deprived areas, and this should inform policy makers regarding organization and delivery of mental health services in Greece and other low-income countries.

## Figures and Tables

**Table 1:** Psychotic patients' characteristics.

	Patients currently in follow-up (*n* = 43)	Disengaged patients (*n* = 21)	Statistical test, *P* value
Age (years, mean, and SD)	50.4 ± 14.5	51.4 ± 15.6	*t* = −0.248, *P* = 0.805
Gender (male)	29	12	*x* ^2^ = 0.65, *P* = 0.42
Illness duration (years, mean, and SD)	22.8 ± 11.5	25.5 ± 11.9	*t* = −0.869, *P* = 0.388
Lives with a caregiver	37	16	*x* ^2^ = 0.963, *P* = 0.326
Substance abuse	15	5	*x* ^2^ = 0.805, *P* = 0.369
Baseline CGI (mean, SD)	4.18 ± 1.18	4.23 ± 1.28	*t* = −0.159, *P* = 0.875
Home-based care	17	9	*x* ^2^ = 0.065, *P* = 0.799
Mean follow-up duration (months, mean, and SD)	31.1 ± 18.77	15.9 ± 13.26	NA

NA: not applicable.
